# Supplementary material for: A gap-filling algorithm for prediction of metabolic interactions in microbial communities
Source: PLoS Comput Biol. 2021 Nov 1;17(11):e1009060. doi: 10.1371/journal.pcbi.1009060 (PMC8584699; doi:10.1371/journal.pcbi.1009060)
Supplement: S1 Appendix — (PDF) [file pcbi.1009060.s001.pdf]

S1 Appendix

Creation of glucose and acetate utilizer strains from the *E. coli* core model

The *E. coli* core model from BiGG [1] was reduced in order to make a model with 72 metabolites and 90 reactions, covering the central carbon metabolism. Performing Flux Balance Analysis (FBA) for our resulting core model, showed that the model grows with a growth rate of 1.26 h^{-1}, while it consumes glucose, oxygen and phosphate at rates 10, 5.4 and 4.6 $mmol \cdot gDW^{-1} \cdot h^{-1}$ respectively. Our resulting core model was the basis for the creation of two *E. coli* strains, one with the ability to use glucose as a substrate and another with the ability to use only acetate excreted from the first strain [2]. In order to create our glucose utilizer, we deleted 5 reactions from the core model. More specifically, we deleted the reaction of phosphoglycerate mutase (PGM), which is the 8th step of glycolysis, the reactions of malate synthase (MALS) and succinyl-CoA synthetase (SUCOAS) fom the TCA cycle, and the reactions of pyruvate formate lyase (PFL) and phosphotransacetylase (PTAr) in pyruvate metabolism. For our acetate utilizer, we deleted the same 5 reactions and 2 more reactions from glycolysis and the TCA cycle: the glucose phosphotransferase system (GLCpts) to impede glucose uptake, and citrate synthase (CS) to limit the channeling of acetyl-CoA. Since acetyl-CoA is a key metabolite through which acetate enters the central metabolism, we knocked out citrate synthase to see whether the model could resume its role as the acetate utilizer in the community despite the deletions around the acetyl-CoA node. Furthermore, we constrained the exchange reactions of the two models by allowing the glucose utilizer to import glucose, oxygen and phosphate, and to import or export acetate, while the acetate utilizer was unable to uptake glucose, but permitted to uptake acetate, oxygen and phosphate. The result of our process is an *E. coli* glucose utilizer model that grows suboptimally, compared to the original core model, and an *E. coli* acetate utilizer that cannot synthesize biomass in the defined conditions based on FBA.

References

1. Orth JD, Fleming RMT, Palsson BØ. Reconstruction and Use of Microbial Metabolic Networks: The Core Escherichia Coli Metabolic Model as an Educational Guide. EcoSal Plus. 2010;4(1). doi:10.1128/ecosalplus.10.2.1.

2. Ho H. Understanding a Methanogenic Benzene-Degrading Culture Using Metabolic Models Created from Metagenomic Sequences [Thesis]. University of Toronto; 2013.
