# Supplementary material for: A gap-filling algorithm for prediction of metabolic interactions in microbial communities
Source: PLoS Comput Biol. 2021 Nov 1;17(11):e1009060. doi: 10.1371/journal.pcbi.1009060 (PMC8584699; doi:10.1371/journal.pcbi.1009060)
Supplement: S2 Appendix — (PDF) [file pcbi.1009060.s002.pdf]

S2 Appendix

Reconstruction and individual curation of the metabolic models of *Dehalobacter* sp. CF and *Bacteroidales* sp. CF50

The metabolic models for the *Dehalobacter* sp. CF (accession no. NC_018866) [1] and the *Bacteroidales* sp. CF50 (accession no. CP006772) were reconstructed automatically by ModelSEED [2] with the use of RAST server [3–5]. Subsequently, the model of *Dehalobacter* sp. CF was validated against its GenBank annotation and an in-house collection of manually curated sequences from the ACT-3 community, while the model of *Bacteroidales* sp. CF50 was verified with its GenBank and KEGG annotations. Then, the models were curated individually, and reactions derived solely from hypothetical proteins, protein domains, or enzymes with ambiguous substrates were excluded from both models.

The metabolic model reconstruction for the *Dehalobacter* sp. CF was curated individually with hydrogen as the energy source and gap-filled in two media that are known to support the microorganism's growth [6–9]: one with acetate, arginine, histidine, and threonine, and another with malate as the only carbon source. Missing biomass precursors were resolved manually by examining their biosynthesis or exchange pathways and adding the necessary reactions [1]. In the *Dehalobacter* sp. CF model dehalogenation is represented by two ferredoxin-mediating reactions, which are coupled to hydrogen oxidation and the reduction of chloroform.

The metabolic model reconstruction for the *Bacteroidales* sp. CF50 was curated individually with lactate as the sole energy and carbon source. Because the *Bacteroidales* strain has not yet been isolated in the lab and the metabolic gaps of its model span across many functional categories, the model was partially curated by adding biochemical reactions from the ModelSEED database to pathways that are at least 60% complete according to genome annotations. Specifically, we assumed that the bacterium could synthesize the standard membrane and cell wall components, including glycerolipids, glycerophospholipids, lipopolysaccharide, and peptidoglycan. Moreover, the manual curation of the model enabled the *de novo* production of histidine, lysine, and NAD, which could not be made due to missing genes in the pathways imidazoleglycerol-phosphate dehydratase (EC: 4.2.1.19) for histidine, 4-hydroxy-tetrahydrodipicolinate synthase (EC: 4.3.3.7) and reductase (EC: 1.17.1.8) for lysine, and nicotinate riboside kinase (EC: 2.7.1.173) and aspartate dehydrogenase (EC: 1.4.1.21) for NAD. After the manual curation, the model was still unable to synthesize several essential metabolites and biomass, mainly due to its incomplete TCA cycle. As a result, alpha ketoglutarate could not be produced directly from the carbon source, and consequently the synthesis of glutamate, glutamine, arginine, proline, and pyridoxal phosphate (vitamin B6) was blocked. The biosynthetic pathways of alanine, tryptophan, quinones, folate-derived metabolites (such as tetrahydrofolate), riboflavin, and heme were also incomplete. However, the organism can potentially acquire many of the needed metabolites via transporters from its environment. For this reason, transporters of amino acids and five organic acids including pyruvate, acetate, malate, fumarate, and succinate were added to the model.

References

1. Correia K, Ho H, Mahadevan R. Genome-Scale Metabolic Network Reconstruction of the Chloroform-Respiring Dehalobacter Restrictus Strain CF. bioRxiv. 2018; p. 375063. doi:10.1101/375063.

2. Seaver SMD, Liu F, Zhang Q, Jeffryes J, Faria JP, Edirisinghe JN, et al. The ModelSEED Biochemistry Database for the Integration of Metabolic Annotations and the Reconstruction, Comparison and Analysis of Metabolic Models for Plants, Fungi and Microbes. Nucleic Acids Research. 2021;49(D1):D575–D588. doi:10.1093/nar/gkaa746.

3. Aziz RK, Bartels D, Best AA, DeJongh M, Disz T, Edwards RA, et al. The RAST Server: Rapid Annotations Using Subsystems Technology. BMC Genomics. 2008;9(1):75. doi:10.1186/1471-2164-9-75.

4. Overbeek R, Olson R, Pusch GD, Olsen GJ, Davis JJ, Disz T, et al. The SEED and the Rapid Annotation of Microbial Genomes Using Subsystems Technology (RAST). Nucleic Acids Research. 2014;42(D1):D206–D214. doi:10.1093/nar/gkt1226.

5. Brettin T, Davis JJ, Disz T, Edwards RA, Gerdes S, Olsen GJ, et al. RASTtk: A Modular and Extensible Implementation of the RAST Algorithm for Building Custom Annotation Pipelines and Annotating Batches of Genomes. Scientific Reports. 2015;5(1):8365. doi:10.1038/srep08365.

6. Grostern A, Duhamel M, Dworatzek S, Edwards EA. Chloroform Respiration to Dichloromethane by a Dehalobacter Population. Environmental Microbiology. 2010;12(4):1053–1060. doi:10.1111/j.1462-2920.2009.02150.x.

7. Grostern A, Edwards EA. A 1,1,1-Trichloroethane-Degrading Anaerobic Mixed Microbial Culture Enhances Biotransformation of Mixtures of Chlorinated Ethenes and Ethanes. Applied and Environmental Microbiology. 2006;72(12):7849–7856. doi:10.1128/AEM.01269-06.

8. Grostern A, Edwards EA. Characterization of a Dehalobacter Coculture That Dechlorinates 1,2-Dichloroethane to Ethene and Identification of the Putative Reductive Dehalogenase Gene. Applied and Environmental Microbiology. 2009;75(9):2684–2693. doi:10.1128/AEM.02037-08.

9. Wang PH, Correia K, Ho HC, Venayak N, Nemr K, Flick R, et al. An Interspecies Malate–Pyruvate Shuttle Reconciles Redox Imbalance in an Anaerobic Microbial Community. The ISME Journal. 2019;13(4):1042–1055. doi:10.1038/s41396-018-0333-4.
