# Supplementary material for: A gap-filling algorithm for prediction of metabolic interactions in microbial communities
Source: PLoS Comput Biol. 2021 Nov 1;17(11):e1009060. doi: 10.1371/journal.pcbi.1009060 (PMC8584699; doi:10.1371/journal.pcbi.1009060)
Supplement: S3 Appendix — (PDF) [file pcbi.1009060.s003.pdf]

S3 Appendix

Reformulation of the community gap-filling algorithm

In the paper, the reaction fluxes of each organism compartment in the community are reported per gram dry weight of the cells of the respective organism. In order to achieve correct mass balance in the community, we can multiply the reaction fluxes of each organism by its relative abundance in the microbial community, and scale the fluxes to one unit of total community biomass. Then, the mathematical formulation of our community gap-filling algorithm resembles that of SteadyCom [1], and our MILP problem for a microbial community of N organisms is written as:

$$\text{minimize} \sum_{n \in N} \sum_{j \in J^n_{\text{Database}}} y^n_j \tag{1}$$

subject to

$$\left[\begin{array}{ll} \sum_{j \in J^n} S^n_{ij}\, f^n\, v^n_j = 0, & \forall\, i \in I^n \\ f^n\, lb^n_j \leq f^n\, v^n_j \leq f^n\, ub^n_j, & \forall\, j \in J^n_{\text{Model}} \\ y^n_j\, f^n\, lb^n_j \leq f^n\, v^n_j \leq y^n_j\, f^n\, ub^n_j, & \forall\, j \in J^n_{\text{Database}} \\ v^n_{\text{Biomass}} \geq f^n\, v^{\text{n, min}}_{\text{Biomass}} & \\ y^n_j \in \{0,1\}, & \forall\, j \in J^n_{\text{Database}} \end{array}\right] \forall\, n \in N \tag{2–6}$$

$$-\, v^c_{\text{ex(i)}} + \sum_{n \in N} f^n\, v^n_{\text{ex(i)}} = 0, \qquad \forall\, i \in I^c \tag{7}$$

$$\sum_{n \in N} f^n = 1\,, \tag{8}$$

where I^n and J^n represent the number of metabolites and reactions, respectively, in the nth microorganism compartment, while I^c represents the exchanged metabolites in the common metabolite pool. The variable v represents reaction fluxes, and f^n is the relative abundance of the organism n in the microbial community.
As mentioned in the Materials and methods section of the paper, Eq (1) is the objective function of the MILP problem which minimizes the total number of biochemical reactions that are added from the database to the metabolic models of the organisms composing the community, and Eq (2) - Eq (7) are the constraints of the optimization problem. Eq (8) indicates that the relative abundances of all the organisms in the microbial community sum up to one.

We applied our reformulated community gap-filling algorithm to our toy *E. coli* and ACT-3 communities, and we compared the results with those obtained from the initial formulation of the community gap-filling algorithm. For the toy *E. coli* community, we assumed that the relative abundance of both strains in the community was 0.5. The first solution calculated by the reformulated community gap-filling algorithm suggested the addition of one reaction to the *E. coli* glucose utilizer model and two reactions to

the *E. coli* acetate utilizer model, as in the Results section of the paper (S29 Table). The difference was that the reaction added to the model of the *E. coli* glucose utilizer was aldehyde dehydrogenase (ALDD2x) that produces acetate from acetaldehyde instead of pyruvate oxidase (POX). The reactions added to the model of the *E. coli* acetate utilizer include the initially knocked out citrate synthase (CS) as in the paper, and xylulose-5-phosphate utilizing phosphoketolase (PKETX), which converts acetyl-phosphate to xylulose-5-phosphate instead of fructose-6-phosphate utilizing phosphoketolase (PKETF). According to the ten best solutions calculated by the reformulated community gap-filling algorithm, all the solutions predicted the ability of the *E. coli* glucose utilizer model to uptake glucose and produce acetate that is consumed by the *E. coli* acetate utilizer model mainly for anaerobic growth (S30 Table), and all the reactions that were added from the database to the community carried realistic fluxes (S31 Table).

In the case of the ACT-3 community, we used the relative abundances of 0.7 and 0.3 for the *Dehalobacter* and the *Bacteroidales* species of the community, respectively. The first solution of the reformulated community gap-filling algorithm (S32 Table) added the reaction of potassium transport via the ABC system (Kabc) to the model of *Dehalobacter* sp. CF, which is used for ATP generation in the exact same way as the reaction of calcium transport via the ABC system (CA2abc) from the paper. Moreover, the reaction anthranilate synthase (ANS2) was added to the model of *Bacteroidales* sp. CF50 in order to produce pyruvate from chorismate, exactly as in the Results section of the paper. The fluxes from the ten best solutions calculated by the reformulated algorithm for the exchange reactions of the community (S33 Table) consistently demonstrated all the experimentally expected metabolic functions of the community [2], and predicted the transfer of CO_2 from the model of *Bacteroidales* sp. CF50 to the model of *Dehalobacter* sp. CF. Regarding the amino acid exchanges, the algorithm predicted consistently the transfer of isoleucine, phenylalanine, and threonine only from *Bacteroidales* sp. CF50 to *Dehalobacter* sp. CF, and the transfer of alanine and glutamate from *Dehalobacter* sp. CF to *Bacteroidales* sp. CF50. Finally, the ten best solutions of the reformulated community gap-filling algorithm (S34 Table) showed that the reactions added to the model of *Dehalobacter* sp. CF carry unrealistically high fluxes, similarly with the Results section of the paper.

Overall, the community gap-filling algorithm when reformulated for taking into account the relative abundances of the organisms in the microbial community, suggested the addition of a few different reactions to the models of the toy *E. coli* and the ACT-3 communities, and it also predicted the transfer of less amino acids from the model of *Bacteroidales* sp. CF50 to the model of *Dehalobacter* sp. CF in the ACT-3 community. However, the number and functionalities of the added reactions, as well as the predicted patterns for metabolite interactions in the communities, remained the same in the solutions calculated by both the reformulated and the initial versions of the community gap-filling algorithm. Therefore, even though the introduction of organism relative abundances in the community gap-filling algorithm affects the results quantitatively and should be preferred as it corrects the mass balance for the metabolites in the community, it does not have a significant effect on the qualitative predictions of community gap-filling.

References

1. Chan SHJ, Simons MN, Maranas CD. SteadyCom: Predicting Microbial Abundances While Ensuring Community Stability. PLOS Computational Biology. 2017;13(5):e1005539. doi:10.1371/journal.pcbi.1005539.

2. Wang PH, Correia K, Ho HC, Venayak N, Nemr K, Flick R, et al. An Interspecies Malate–Pyruvate Shuttle Reconciles Redox Imbalance in an Anaerobic Microbial Community. The ISME Journal. 2019;13(4):1042–1055. doi:10.1038/s41396-018-0333-4.
