# Supplementary figures and images for: A gap-filling algorithm for prediction of metabolic interactions in microbial communities
Source: PLoS Comput Biol. 2021 Nov 1;17(11):e1009060. doi: 10.1371/journal.pcbi.1009060 (PMC8584699; doi:10.1371/journal.pcbi.1009060)

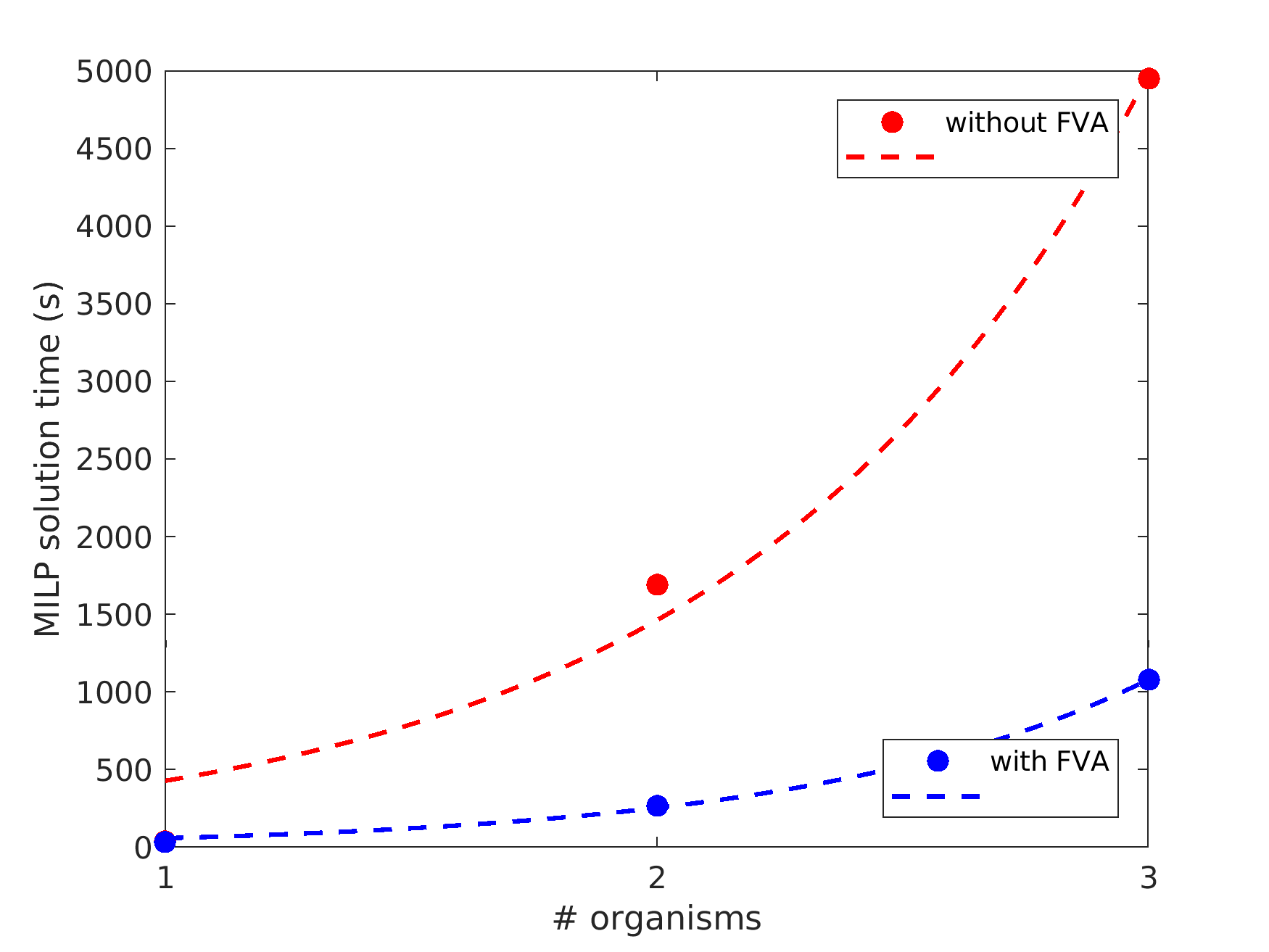

Supplement: S1 Fig — As the number of organisms that make up the microbial community increases, the community gap-filling algorithm needs increasingly more time to solve the MILP problem that restores growth in the community. However, the increase in solving time is significantly reduced with the use of our community gap-filling method that reduces the solution space of the MILP problem by performing FVA for each organism compartment of the community before formulating the optimization problem. (The presented measurements were performed with the models from the toy E. coli community). (TIF) [file pcbi.1009060.s042.tif]

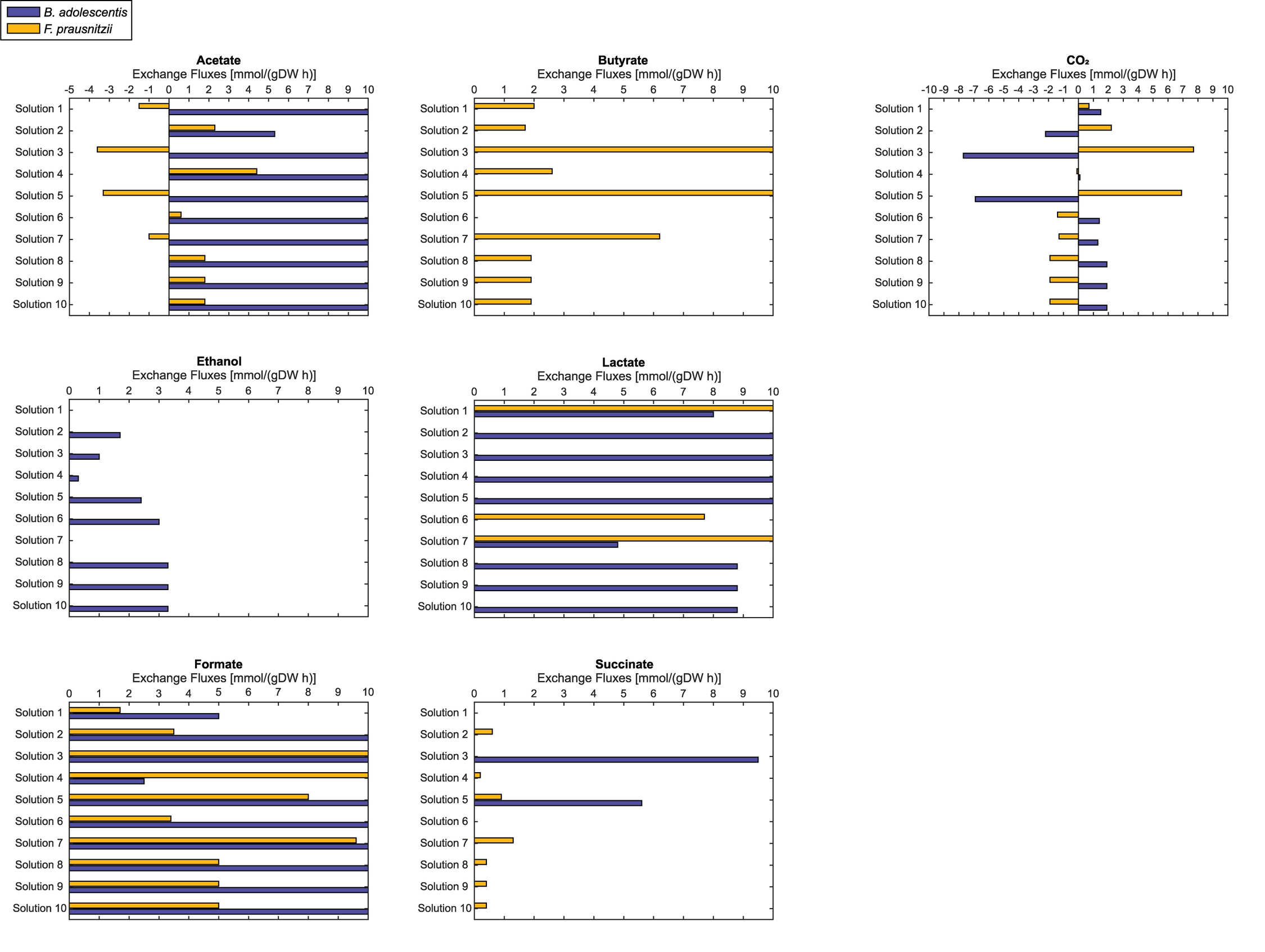

Supplement: S2 Fig — Fluxes of exchange reactions for the SCFAs acetate, butyrate, ethanol, lactate, formate, and succinate, and CO2. (TIF) [file pcbi.1009060.s043.tif]

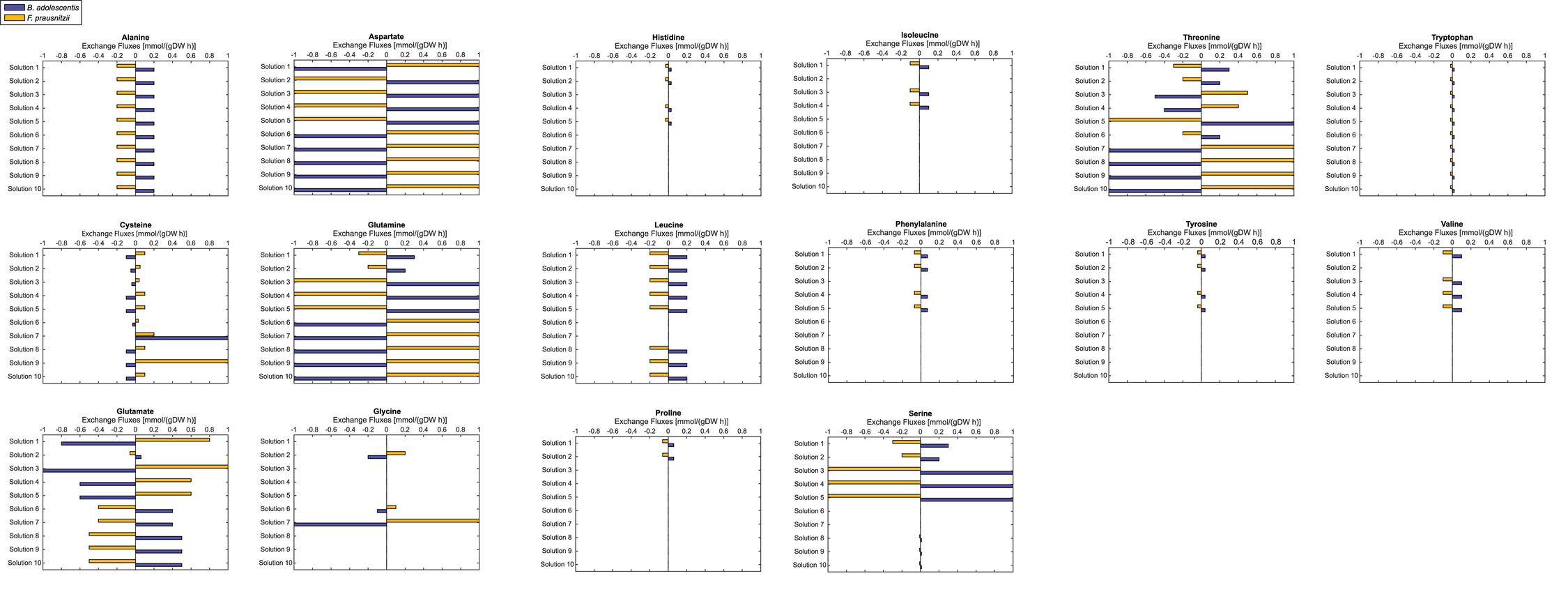

Supplement: S3 Fig — Fluxes of exchange reactions for the amino acids alanine, aspartate, cysteine, glutamine, glutamate, glycine, histidine, isoleucine, leucine, phenylalanine, proline, serine, threonine, tryptophan, tyrosine, and valine. (TIF) [file pcbi.1009060.s044.tif]

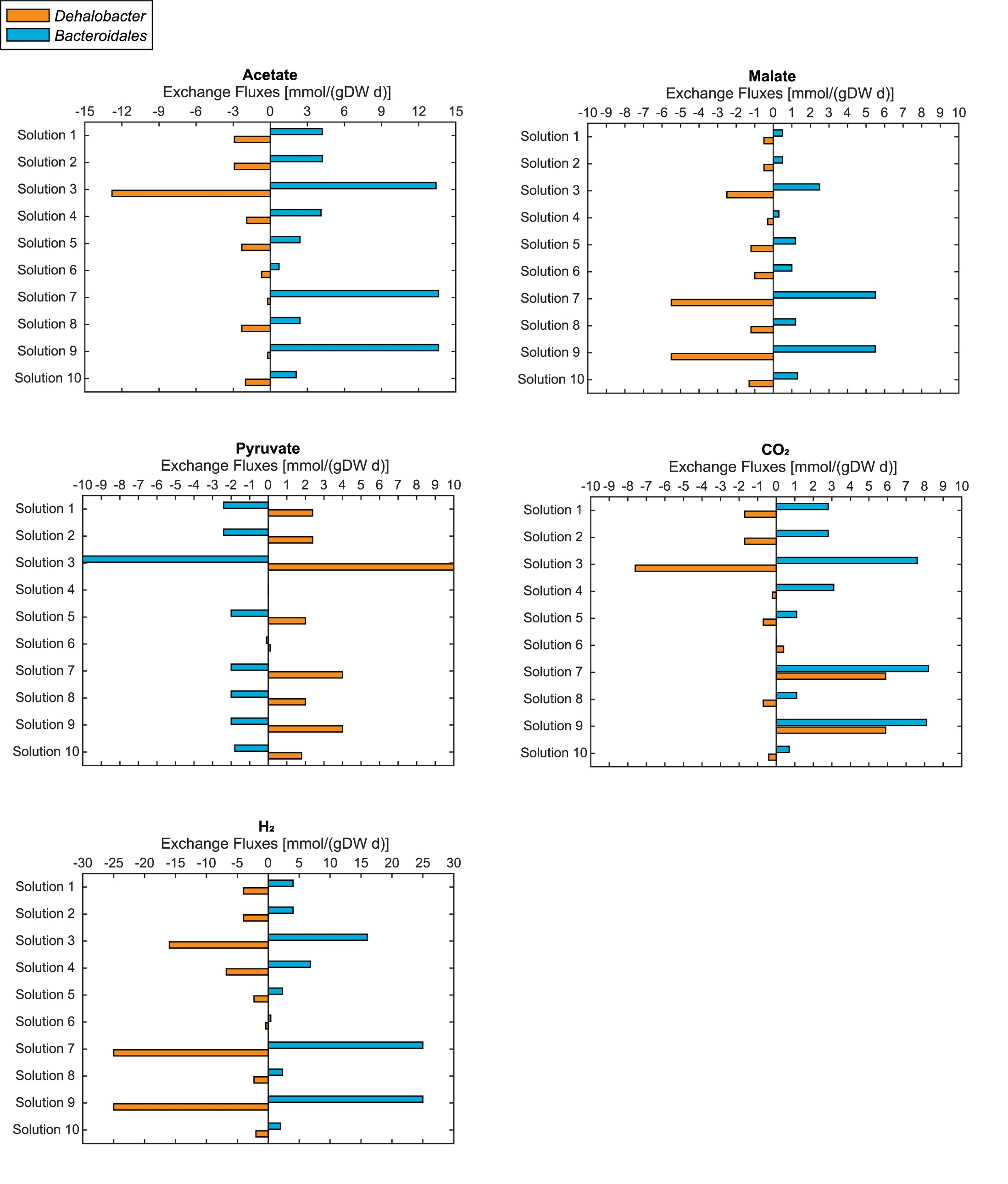

Supplement: S4 Fig — Fluxes of exchange reactions for the organic acids acetate, malate, and pyruvate, CO2 and H2. (TIF) [file pcbi.1009060.s045.tif]

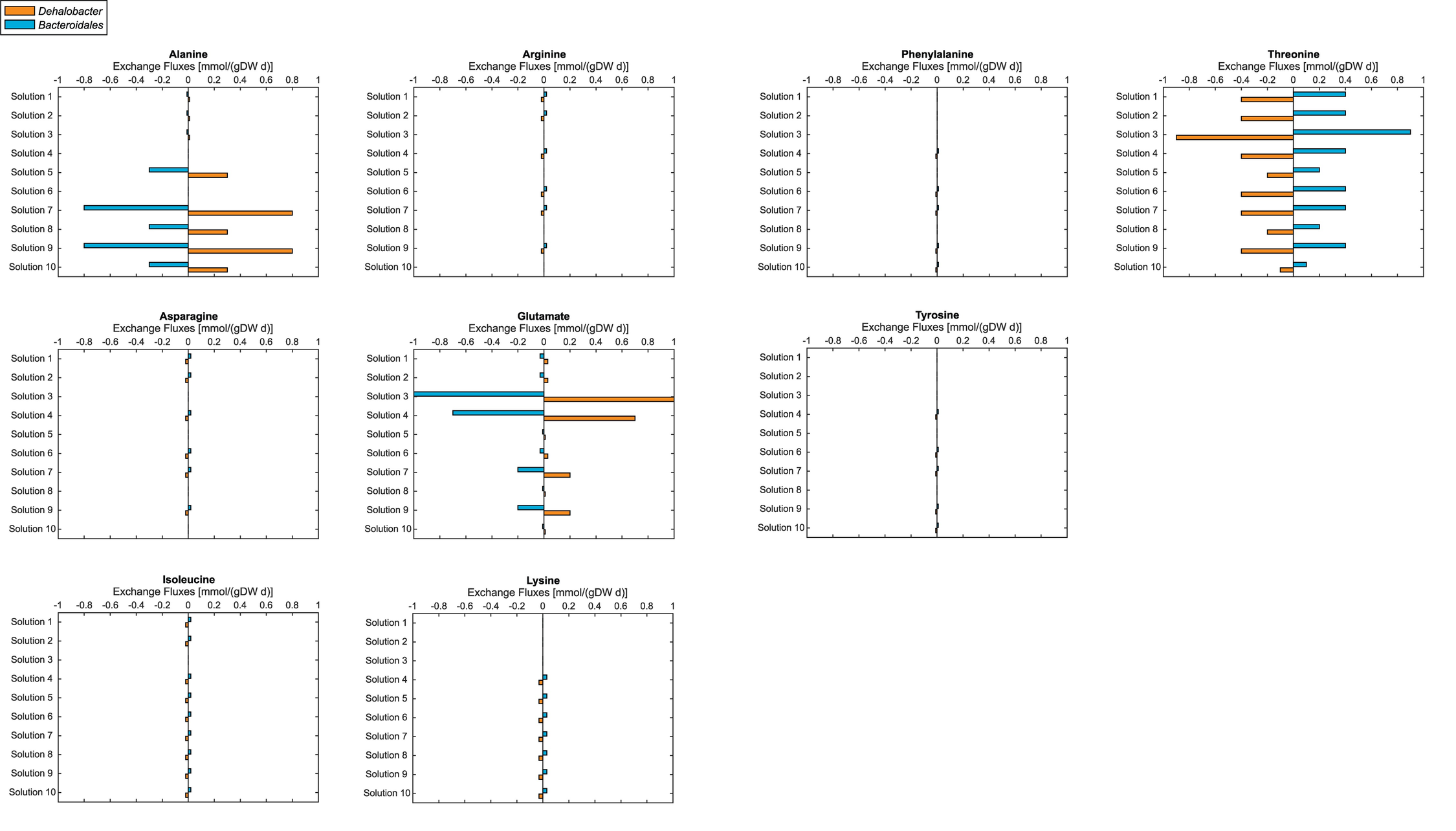

Supplement: S5 Fig — Fluxes of exchange reactions for the amino acids alanine, arginine, asparagine, glutamate, isoleucine, lysine, phenylalanine, threonine, and tyrosine. (TIF) [file pcbi.1009060.s046.tif]
